# A Novel Laccase with Potent Antiproliferative and HIV-1 Reverse Transcriptase Inhibitory Activities from Mycelia of Mushroom *Coprinus comatus*

**DOI:** 10.1155/2014/417461

**Published:** 2014-08-28

**Authors:** Shuang Zhao, Cheng-Bo Rong, Chang Kong, Yu Liu, Feng Xu, Qian-Jiang Miao, Shou-Xian Wang, He-Xiang Wang, Guo-Qing Zhang

**Affiliations:** ^1^Institute of Plant and Environment Protection, Beijing Academy of Agriculture and Forestry Sciences, Beijing Engineering Research Center for Edible Mushroom, Beijing 100097, China; ^2^State Key Laboratory for Agrobiotechnology and Department of Microbiology, China Agricultural University, Beijing 100193, China; ^3^Key Laboratory of Urban Agriculture (North) of Ministry of Agriculture, College of Biological Sciences and Engineering, Beijing University of Agriculture, Beijing 102206, China

## Abstract

A novel laccase was isolated and purified from fermentation mycelia of mushroom *Coprinus comatus* with an isolation procedure including three ion-exchange chromatography steps on DEAE-cellulose, CM-cellulose, and Q-Sepharose and one gel-filtration step by fast protein liquid chromatography on Superdex 75. The purified enzyme was a monomeric protein with a molecular weight of 64 kDa. It possessed a unique N-terminal amino acid sequence of AIGPVADLKV, which has considerably high sequence similarity with that of other fungal laccases, but is different from that of *C. comatus* laccases reported. The enzyme manifested an optimal pH value of 2.0 and an optimal temperature of 60^°^C using 2,2′-azinobis(3-ethylbenzothiazolone-6-sulfonic acid) diammonium salt (ABTS) as the substrate. The laccase displayed, at pH 2.0 and 37^°^C, *K* *_m_* values of 1.59 mM towards ABTS. It potently suppressed proliferation of tumor cell lines HepG2 and MCF7, and inhibited human immunodeficiency virus type 1 (HIV-1) reverse transcriptase (RT) with an IC_50_ value of 3.46 *μ*M, 4.95 *μ*M, and 5.85 *μ*M, respectively, signifying that it is an antipathogenic protein.

## 1. Introduction

Laccases (benzenediol:oxygen oxidoreductase; EC 1.10.3.2), belonging to polyphenol oxidases, play a key role in lignin degradation in nature. They can oxidate a variety of phenolic and inorganic compounds, including diphenols, polyphenols, and substituted phenols, using molecular oxygen as the electron acceptor [1, 2]. Although they were first reported and named from plant, laccases are widely distributed in higher plants and fungi and have also been found in insects and bacteria [3]. Laccases are involved in various physiological roles in nature. Botanical laccases contribute to lignin synthesis, while fungal laccases are conversely involved in lignin degradation and also pathogenesis [3, 4]. In recent years, the occurrence and properties of the laccases have been comprehensively reviewed due to their potential uses in lignin biodegradation, synthetic dye decolorization, paper-pulp bleaching, bioremediation, biosensors, chemical synthesis, and so forth [2, 5].

*Coprinus comatus*, commonly named as lawyer's wig or shaggy mane, is a common fungus often seen growing on lawns in spring or autumn. In China, Japan, and other Asian countries, the species is cultivated as an excellent edible mushroom. It is very unusual because it is edible when young but becomes poisonous when old. The cap is white and closely covers the stipe over; then it turns black and dissolves itself in a matter of hours after being picked or depositing spores. Previous studies on* C. comatus* mainly focus on its polysaccharide extract, antitumor and immunomodulatory activities, fermentation, and so forth. In recent researches, ethyl acetate extract from fruit bodies of* C. comatus* manifested antiproliferative activity towards human ovarian cancer cell lines [6]. A water-soluble polysaccharide demonstrated inhibitory activity towards Sarcoma 180 tumor cell in mice. It can significantly enhance the Con A- or LPS-induced splenocyte proliferation and increase the production of TNF-*α* and IL-2 [7]. Vanadium-enriched* C. comatus* promoted femoral fracture healing in streptozotocin-diabetic rats with a 35.5% increase in the total area of callus [8]. There are only few literatures about bioactive proteins from* C. comatus*. A fibrin-specific fibrinolytic enzyme was produced by liquid culture of* C. comatus* [9]. Recently, Bao et al. cloned a laccase isoenzyme gene from* C. comatus*, and functionally heterologously expressed it in* Pichia pastoris* [10].

In the present work, preliminary studies based on mycelia of* C. comatus* showed that laccase extract demonstrated different enzymatic properties from those of the recombinant laccase newly reported [10]. Hence, we aim to purify the laccase from* C. comatus* and then study its properties and applications.

## 2. Materials and Methods

### 2.1. Strain and Culture Condition

Fruiting bodies of* C. comatus* were collected in the campus of China Agricultural University (Beijing, China). Strain JT-01 was isolated from fresh fruiting bodies. Strain identification was based on a standard ITS sequence amplification and analysis and also fruiting experiments [11]. The fungus was cultured at 26°C, stored at 4°C, and monthly transferred to fresh PDA slants which contained (g/L) potato, 200; glucose, 20; and agar, 20. For purification of the laccase, strain JT-01 was inoculated into the liquid PD media which contained (g/L) potato, 200, and glucose, 20. The media were cultured using an orbital shaking incubator at 200 rpm and 26°C for 2 weeks. Then, the mycelia were collected for further laccase purification.

### 2.2. Assay for Laccase Activity

Laccase activity was determined by a modified method described by Shin and Lee using 2,2′-azinobis(3-ethylbenzothiazolone-6-sulfonic acid) diammonium salt (ABTS) as the substrate [12]. In brief, enzyme solution (5 *μ*L) was mixed with 1 mM ABTS solution (145 *μ*L, in 50 mM sodium acetate buffer, pH 4.5) at 37°C for 5 min, followed by ending the reaction by an addition of 10% TCA (250 *μ*L). The change in the absorbance was monitored at 405 nm for enzyme activity. One enzyme unit (U) was defined as the amount of enzyme required to produce one absorbance increase at 405 nm per minute per millilitre of the reaction mixture under the assay conditions. All determinations were performed in triplicate.

### 2.3. Purification of Laccase

After two-week fermentation, mycelia were harvested by centrifugation at 10000 rpm and 4°C for 15 min. Subsequently, the mycelia homogenized and extracted in 0.15 M NaCl (1 : 4, w/v) at 4°C overnight, followed by another centrifugation at 10000 rpm and 4°C for 15 min. Then, (NH_4_)_2_SO_4_ was added to the supernatant until 80% saturation to precipitate proteins. The mixture was left 4°C for 4 h, then centrifuged at 10000 rpm and 4°C for 15 min, and dialyzed against distilled water overnight. The crude laccase extract was further purified by three successive steps of ion exchange chromatography: firstly on DEAE-cellulose (10 mM phosphate buffer, pH 7.0) with a flow rate of 2 mL/min, secondly on CM-cellulose (10 mM phosphate buffer, pH 6.6) with a flow rate of 2 mL/min, and finally on Q-Sepharose (10 mM sodium acetate buffer, pH 4.0) with a flow rate of 1 mL/min. The laccase active fraction was finally purified by fast protein liquid chromatography (FPLC) on a Superdex 75 HR 10/30 gel filtration column (0.2 M NH_4_HCO_3_ buffer, pH 8.5) with a flow rate of 0.8 mL/min.

### 2.4. Determination of Molecular Mass

To determine the molecular mass (*Mr*) of the purified laccase, both FPLC-gel filtration and sodium dodecyl sulfate-polyacrylamide gel electrophoresis (SDS-PAGE) were used. In FPLC-gel filtration, a standard curve based on elution volume and* Mr* of molecular mass standards (GE Healthcare) can be obtained.* Mr* of the present laccase fraction can be calculated [15]. In SDS-PAGE, a 12% resolving gel and a 5% stacking gel were used following procedure of Laemmli and Favre [16]. The* Mr* was calculated based on another curve of relative mobility and log* Mr*.

### 2.5. Determination of N-Terminal Amino Acid Sequence

The N-terminal amino acid sequence of the purified laccase was determined using a Hewlett-Packard HP G1000A Edman degradation unit and an HP 1000 HPLC System [17].

### 2.6. Effect of pH and Temperature on Laccase Activity

In the pH assay, a series ABTS solution in different pH value was used instead of the ABTS solution at pH 4.5 in the standard enzyme assay. The assay buffers were prepared in KCl-HCl buffers (pH 1.1–2.2) and sodium citrate acid buffers (pH 2.2–8.0). In the temperature assay, standard assay mixture was tested in different temperature (20–100°C) instead of 37°C in the standard assay.

### 2.7. Assay for Enzyme Kinetic of Purified Laccase

The Michaelis-Menten constants of the purified laccase were determined using ABTS as substrate at pH 2.0 in various concentrations (0.5, 1.0, 1.5, 2.0, 3.0, 4.0, and 5.0 mM) and at 37°C. All determinations were performed in triplicate. The* Km* values were obtained from a Lineweaver-Burk plot [24].

### 2.8. Effect of Metal Ions and EDTA on Laccase Activity

To estimate metal ions and EDTA on enzyme activity, equal volumes of the purified laccase solution were preincubated with metal ions or EDTA solutions (at concentrations of 2.5, 5.0, 10, and 20 mM, resp.) at 4°C for 1 h before the standard laccase assay was performed. The chemical reagents of metal ions were including AlCl_3_, CaCl_2_, CoCl_2_, CuCl_2_, FeCl_2_, HgCl_2_, KCl, LiCl, MgCl_2_, MnCl_2_, ZnCl_2_, and EDTA. Control samples were assayed without the metal ions. All determinations were performed in triplicate.

### 2.9. Assay of Antiproliferative Activity

Antiproliferative activity has been reported for many mushroom proteins [25]. The tumor cell lines human breast cancer (MCF7) and hepatoma (HepG2) were purchased from American Type Culture Collection (ATCC). HepG2 cells were cultured in Dulbecco modified Eagle's medium (DMEM), and MCF7 cells were cultured in RPMI medium supplemented with 10% (v/v) fetal bovine serum (FBS), 100 mg/l streptomycin, and 100 IU/mL penicillin at 37°C in a humidified atmosphere of 5% (v/v) CO_2_. Cells were seeded into 96-well plates with a concentration of 8 × 10^3^ cells/well and incubated for 24 h. Different concentrations of* C. comatus* laccase were added into the wells with serum-free medium and incubated for 72 h. After that, MTT assays were carried out to measure cell viability. Briefly, 20 *μ*L of a 5 mg/mL solution of MTT in serum-free medium was spiked into each well, and the plates were incubated for 4 h. The supernatant was carefully removed, and 200 *μ*L of DMSO was added into each well to dissolve the MTT formazan. The absorbance at 560 nm was measured with a microplate reader. PBS was added into the wells instead of laccase as the control [11, 26].

### 2.10. Assay for HIV-1 Reverse Transcriptase Inhibitory Activity

The assay for the inhibitory activity on human immunodeficiency virus type 1 (HIV-1) reverse transcriptase (RT) was assessed using an enzyme-linked immunosorbent assay (ELISA) kit from Boehringer (Mannheim, Germany) [27]. The assay takes advantage of the ability of reverse transcriptase to synthesize DNA, starting from the template primer hybrid poly(A) oligo(dT)15. An optimized ratio of the digoxigenin- and biotin-labeled nucleotides is incorporated into one of the DNA molecules synthesized by the RT. The detection and quantification of synthesized DNA as a parameter for RT activity follow the sandwich ELISA protocol. Biotin-labeled DNA binds to the surface of microtiter plate modules precoated with streptavidin. An antibody to digoxigenin conjugated to peroxidase (anti-DIG-POD) binds to the digoxigenin-labeled DNA. Finally, the peroxidase substrate is added. The peroxidase enzyme catalyzes the cleavage of the substrate and produces a colored reaction product. The absorbance of the samples at 405 nm can be determined by using a microtiter plate (ELISA) reader and is directly correlated with the level of RT activity. A fixed amount (4–6 ng) of recombinant HIV-1 RT was used. The inhibitory activity of the laccase was calculated as percent inhibition compared to a control without the protein.

## 3. Results

### 3.1. Laccase Purification and Molecular Mass Determination

**Figure 1:**
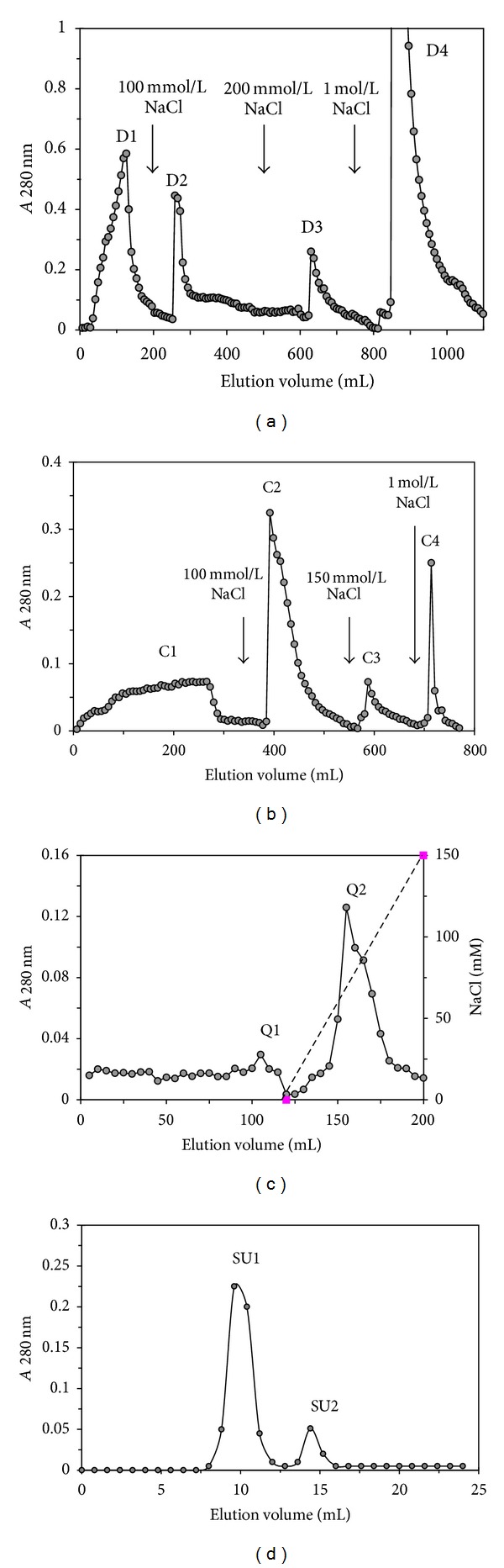
Elution profiles of* C. comatus* laccase. (a) Ion exchange chromatography on DEAE-cellulose column. Fraction D3 was the laccase concentrated fraction. (b) Ion exchange chromatography on CM-cellulose column. Fraction C3 was the laccase concentrated fraction. (c) Ion exchange chromatography on Q-Sepharose column. Fraction Q2 was the laccase concentrated fraction. (d) Gel filtration on Superdex 75. Fraction SU1 was purified laccase.

**Figure 2:**
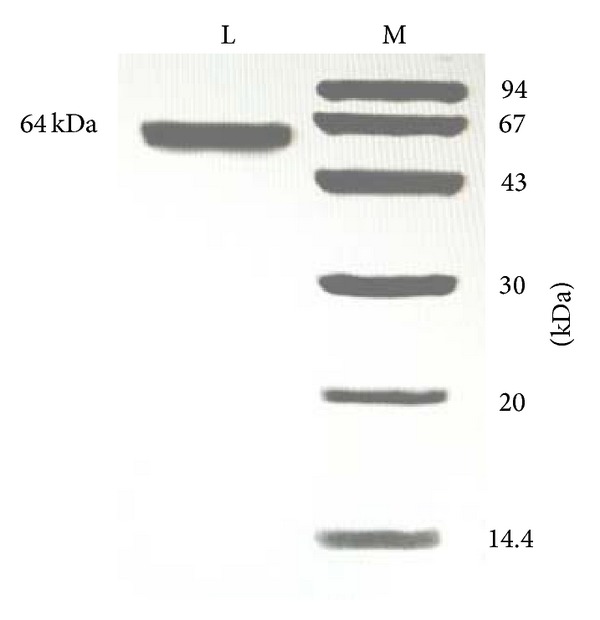
SDS-PAGE of* C. comatus* laccase (fraction SU1). Left lane:* C. comatus* laccase. Right lane: molecular mass markers. From top downward: phosphorylase b (94 kD), bovine serum albumin (67 kD), ovalbumin (43 kD), carbonic anhydrase (30 kD), soybean trypsin inhibitor (20 kD), and lactalbumin (14.4 kD).

The laccase was highly purified from the cultured mycelial extract by employing initial ammonium sulfate precipitation and centrifugation steps, followed by three ion-exchange chromatography steps on DEAE-cellulose, CM-cellulose, and Q-Sepharose and a final gel-filtration step by fast protein liquid chromatography on Superdex 75. The yields and specific laccase activities at various stages of purification are listed in Table 1. An overall 52.6-fold purification was achieved with an activity recovery of 7.8%. The crude enzyme extract was chromatographed on DEAE-cellulose into four fractions: D1, D2, D3, and D4 after elution with 0, 100, 200, and 1000 mM NaCl, respectively, in phosphate buffer (10 mM, pH 7.0) (Figure 1(a)). The fraction D3 with laccase activity was subsequently applied to CM-cellulose and eluted with phosphate buffer (10 mM, pH 6.6). It was also separated into four fractions C1, C2, C3, and C4 with 0, 100, 150, and 1000 mM NaCl in the same buffers, respectively (Figure 1(b)). Fraction C3 containing laccase activity was further fractionated to Q-Sepharose and eluted with a linear gradient of 0–150 mM NaCl in sodium acetate buffer (pH 4.0). It was divided into an unabsorbed fraction Q1 with no laccase activity and a larger absorbed fraction Q2 with high laccase activity (Figure 1(c)). Finally, fraction Q2 was resolved into two peaks with gel filtration on Superdex 75 (Figure 1(d)). Laccase activity was concentrated in fraction SU1 with a molecular mass (*Mr*) of 64 kDa. In SDS-PAGE, SU1 fraction appeared as a single band with a* Mr* of 64 kDa (Figure 2). This suggested that the native laccase is a monomeric protein.

### 3.2. Properties of Purified Laccase

**Figure 3:**
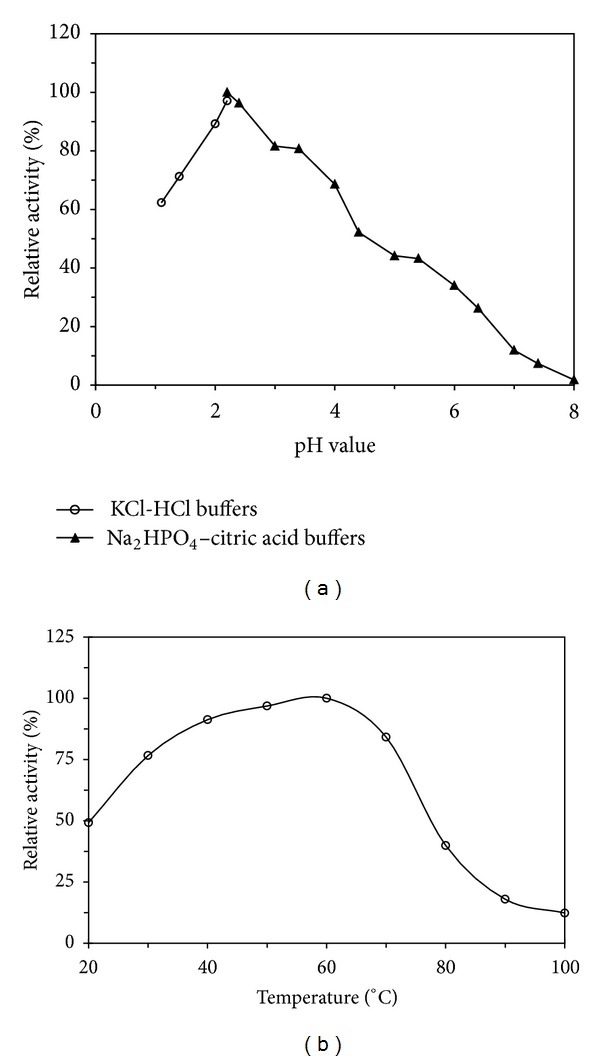
Optimal pH and temperature of* C. comatus* laccase. (a) pH optimum of the purified laccase. Laccase activity was assayed towards ABTS (pH 1.1–8.0) at 37°C. (b) Temperature optimum of the purified laccase. Assay solution was assayed at 20–100°C instead of 37°C in the standard enzyme assay.

**Figure 4:**
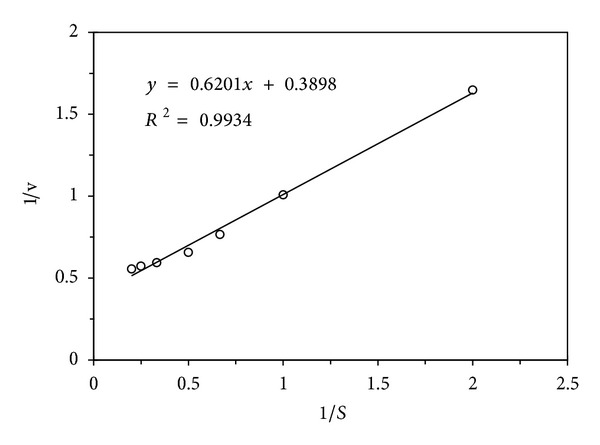
Determination of the kinetics parameter of purified* C. comatus* laccase towards ABTS using Lineweaver-Burk plot.

The N-terminal amino acid sequence of* C. comatus* laccase (CCL) is AIGPVADLKV. An N-terminal amino acid sequence comparison of CCL in the present study and other laccases or laccase-like proteins from* C. comatus* earlier reported was listed in Table 2. Another comparison of CCL and other fungal laccases in their N-terminal amino acid sequences was presented in Table 3. The purified laccase expressed its maximal oxidizing activity towards ABTS at pH 2.0 (Figure 3(a)). It underwent a sharp increase and a continuous decrease in enzyme activity as assayed in pH 1.1–8.0. The purified enzyme possessed a considerable high optimal temperature of 60°C. Oxidizing activity towards ABTS at 60°C was twice as high as that at 20°C and about 1.2 times as high as that at 40°C and 80°C. More than 10% of total enzyme activity remained when it was assayed at 100°C (Figure 3(b)). After incubation of the purified laccase with various ABTS concentrations (0.5–5.0 mM), the reactions are found to follow Michaelis-Menten kinetics, displaying the* Km* value of 1.59 mM towards ABTS using Lineweaver-Burk plots (Figure 4). The sensitivity of CCL to metal ions and EDTA is shown in Table 4. The purified enzyme activity is not significantly affected by the presence of EDTA at an assay concentration range of 1.25–10 mM, K^+^, and Co^2+^ at the assay concentration range of 1.25–5.0 mM, Cu^2+^, Mn^2+^, and Zn^2+^ at the assay concentration range of 1.25–2.5 mM. Cu^2+^ can slightly enhance the enzyme activity of about 10% when the assay concentration reached 5.0–10 mM. On the contrary, CCL activity was continuously reduced by Fe^2+^, Hg^2+^, Ca^2+^, Mn^2+^, Li^+^, and Al^3+^ when the ion concentration rises from 1.25 mM to 10 mM.

**Figure 5:**
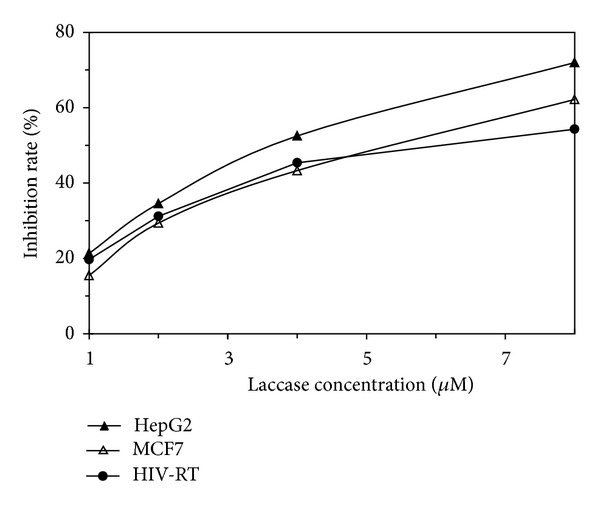
Inhibitory activities of* C. comatus* laccase against HepG2, MCF7, and HIV-1 reverse transcriptase.

Antiproliferative activity towards tumor cell lines and inhibitory activity towards HIV-1 RT were determined using IC_50_ value which is the concentration of IBL that results in an inhibition ratio of 50%. The purified laccase demonstrates antiproliferative activity towards tumor cell lines HepG2 and MCF7 and inhibitory activity towards HIV-1 RT with IC_50_ values of 3.46 *μ*M, 4.95 *μ*M, and 5.85 *μ*M, respectively (Figure 5).

## 4. Discussion

The shaggy mane mushroom is a common but unusual mushroom species because it is widely distributed but will turn black and dissolve itself in a matter of hours after maturation. Up to now, only 6 protein sequences from* C. comatus* have been reported (EMB or GenBank Accession numbers CDJ79885.1, CDJ79884.1, AFD97050.1, AFD97049.1, ABS10994.1, and ABS10993.1) [13]. The present laccase possesses considerably high sequence similarity with those* C. comatus* laccases or laccase-like proteins reported, but they are obviously different with the highest similarity of 80%. It suggests that the purified laccase in the present study is a novel laccase among those* C. comatus* laccases reported. On the other hand, CCL also manifests considerable similarity with other Polyporaceae laccases at the N-terminal position, which suggests that they might share a similar protein secondary or tertiary structure.

During the purification process, CCL was absorbed on DEAE-cellulose, CM-cellulose, and Q-Sepharose, just like other fungal laccases from* Agrocybe cylindracea* [17],* Russula virescens* [28], and* Pleurotus eryngii* [29]. On the other hand, a newly reported laccase from* Lepiota ventriosospora* is unabsorbed on CM-cellulose [30]. The purification factor and yield are related to elution procedure. CCL manifests a purification factor of 52.6-fold, which is considerably higher than that of* Clitocybe maxima* (16.8-fold) [27] and the monkey head mushroom* Hericium erinaceus* (15-fold) [31]. During the purification of* C. maxima* laccase, SP-Sepharose, a kind of strong cation chromatography media, was used instead of CM-cellulose. Parts of laccase active fraction might be irreversibly adsorbed on the gel. On the other hand, when the* H. erinaceum* laccase was purified, four successive steps of ion exchange chromatography and one gel filtration were used. The more the purification steps were used, the less the objective proteins were gained.

The present laccase is a monomeric protein with a molecular mass of 64 kDa, which falls well within the range of molecular masses for most of the fungal laccases reported (50–90 kDa) [3]. It is just the same as that of laccase from* Pleurotus nebrodensis* (64 kDa) [32]. that of laccases from* Pleurotus nebrodensis* (64 kDa) [32],* H. echinaceus* (63 kDa) [31]. Just like most of the fungal laccases,* C. comatus* laccase is a monomeric protein, while laccases from* Phellinus ribis* and* Gaeumannomyces graminis* are dimeric and quadruple proteins, respectively [34, 35].

The purified laccase manifests a quite low pH optimum of pH 2.0 towards ABTS, which is much lower than that of laccases from* A. cylindracea* (pH 5.2) [17],* P. nebrodensis* (pH 5.0) [32], and* L. ventriosospora* (pH 4.0) [30]. Laccases from* A. blazei* (pH 2.3) and* R. virescens* (pH 2.2) also share a very low pH optimum, while laccase from* Pleurotus ostreatus* reaches its maximal oxidizing activity towards ABTS at pH 6.9 which is very close to neutral [23]. The present laccase possesses a considerable high optimal temperature of 60°C, just the same as that of laccases from* R. virescens* [28], twice as that of laccase from* A. placomyces* (30°C) [17], and three times as that of laccase from the sanghuang mushroom* Inonotus baumii* (20°C) [11]. It suggests that CCL manifests a potential application at low pH and high temperature conditions.

The* Km* value of the purified laccase towards ABTS at pH 2.0 and 37°C was 1.59 mM which was 10 times higher than that of* R. virescens* laccase (0.115 mM) [28]. A recombinant expressed* C. comatus* laccase (Lac1) manifested a much lower* Km* value towards ABTS of 34 *μ*M at its optimal enzymatic conditions (pH 3.0 and 65°C) [10]. In the present study, the* Km* value was assayed at a lower temperature of 37°C but not its optimal temperature of 60°C. That is why* Km* of CCL was much higher than that of Lac1. The present laccase was not sensitive to the assayed metal ions and EDTA except Fe^2+^. Fe^2+^ is a reducing agent and strongly decreases most of the reported laccases, such as Lac1 from* C. comatus* [10] and other fungal laccases from* Abortiporus biennis* [36],* I. baumii* [11], and* R. virescens* [28]. Cu^2+^ was a special ion for the laccase activity. High concentration of Cu^2+^ (5–10 mM) slightly increased the laccase activity just like that of laccases for* Polyporus* sp. [37] and* A. biennis* [36]. CCL is not significantly affected by the presence of EDTA (1.25–10 mM). It is suggested that the core metal ions were stable at the concentration.

It is remarkable that some of mushroom components including laccases, lectins, polysaccharopeptides, and antifungal proteins exhibit inhibitory activities towards tumor cells or HIV-1 RT. In the present study, CCL manifests both antiproliferative and anti-HIV-1 RT activities with an IC_50_ value towards HepG2, MCF7, and HIV-1 RT of 3.46 *μ*M, 4.95 *μ*M, and 5.85 *μ*M, respectively, indicating that it is also an antipathogenic protein. The laccase from* I. baumii*, a very famous traditional Chinese medicinal mushroom, also manifests antiproliferative activities towards tumor cell lines HepG2 and L1210 with IC_50_ values of 2.4 *μ*M and 3.2 *μ*M, respectively, but is devoid of inhibitory activity toward HIV-1 RT. On the other hand, the present laccase is purified from liquid fermentation, which means that the protein is very easy to obtain. It is noteworthy that it possesses further applications of agents for cancer or AIDS therapy.

## 5. Conclusions

In summary, a novel laccase (CCL) with a distinctive N-terminal sequence is purified from mycelia of mushroom* C. comatus* obtained from liquid fermentation. Characterization studies show that the enzyme possesses a molecular mass of 64 kDa, a pH optimum at 2.0, a temperature optimum at 60°C, and a *K*_*m*_ value of 1.59 mM towards ABTS. The laccase also exhibits antiproliferative activity towards tumor cells and inhibitory activity toward HIV-1RT, suggesting that it is an antipathogenic protein.

## Figures and Tables

**Table 1:** Yields and laccase activities of various chromatographic fractions (from 50 g mycelia).

Fraction	Yield (mg)	Total activity (U)	Specific activity (U/mg)	Recovery of activity (%)	Purification fold
80% ammonium sulphate fractionation	996	13814.42	13.86	100	1
D1	104.18	—	—	—	—
D2	85.89	—	—	—	—
**D3**	**35.31**	**3357.96**	**95.09**	**24.3**	**6.9**
D4	436.16	—	—	—	—
C1	20.62	—	—	—	—
C2	14.67	—	—	—	—
**C3**	**3.234**	**2091.72**	**646.79**	**15.1**	**46.6**
C4	2.28	—	—	—	—
Q1	1.76	—	—	—	—
**Q2**	**1.85**	**1334.11**	**721.14**	**9.7**	**52**
**SU1**	**1.48**	**1078.97**	**729.04**	**7.8**	**52.6**
SU2	0.35	—	—	—	—

—: no laccase activity observed. Laccase-enriched fractions were highlighted in boldface.

**Table 2:** Comparison of partial amino acid sequence of *C. comatus* laccase (CCL) in this study and other laccase or laccase-like proteins from *C. comatus* earlier reported.

Protein name	Partial amino acid sequence	Reference
CCL	1 **AIGPV ADLKV** 10	This study
Laccase 1 (AFD97050.1)	19 **AIGPV** **ADLHI** 28	[10]
Laccase 2 (AFD97049.1)	22 **AIGPN ADLFI** 31	[10]
Benzenediol:oxygen oxidoreductase (CDJ79885.1)	18 **SVGPR ATLTL** 27	[13]
Benzenediol:oxygen oxidoreductase (CDJ79884.1)	28 **VLIPH STLTL** 37	[13]
Laccase-like multicopper oxidase (ABS10994.1)	Not match	[14]
Laccase-like multicopper oxidase (ABS10993.1)	Not match	[14]

Amino acid residues identical to the corresponding residues of the purified laccase are underlined.

**Table 3:** Comparison of the N-terminal sequence of *C. comatus *laccase (CCL) with other fungal laccases.

Fungal laccase	N-terminal sequence	Reference
*Coprinus comatus *laccase (CCL)	**AIGPVADLKVI**	This study
*Pycnoporus cinnabarinus *laccase	**AIGPVADLTL**	[18]
*Trametes versicolor *laccase I	**AIGPVASLVV**	[19]
*Trametes versicolor* laccase II	**GIGPVADLTI**	[19]
*Basidiomycete *PM1 laccase	**SIGPVADLTI**	[20]
*Trametes versicolor *laccase III	**GIGPVADLTI**	[12]
*Coriolus hirsutus *laccase	**AIGPTADLTI**	[21]
*Inonotus baumii* laccase	**AIGPVDEV**	[11]
*Ceriporiopsis subvermispora *laccase	**AIGPVTDLEI**	[22]
*Pleurotus ostreatus *laccase	**AIGPDGNMYI**	[23]

Amino acid residues identical to the corresponding residues of the purified laccase are underlined.

**Table 4:** Effect of metal ions and EDTA on *C. comatus *laccase activity.

Metal ions	Residual activity (% of control)			
	1.25 mM	2.5 mM	5 mM	10 mM
Al^3+^	89.1	88.5	85.4	84.3
Ca^2+^	86.0	83.8	80.6	69.7
Co^2+^	100	100	100.9	89.9
Cu^2+^	98.0	100	110.8	110.4
Fe^2+^	34.2	28.1	22.1	10.7
Hg^2+^	82.3	71.2	61.8	45.5
K^+^	98.0	106.4	97.8	91.0
Li^+^	85.3	86.8	87.4	83.0
Mg^2+^	99.7	100	86.8	80.9
Mn^2+^	94.1	91.1	85.8	70.0
Zn^2+^	100	98.9	90.0	88.0
EDTA	98.9	100	98.0	97.5

Laccase activity in the absence of metal ions was regarded as 100%.

## References

[1] Leontievsky AA, Vares T, Lankinen P (1997). Blue and yellow laccases of ligninolytic fungi. *FEMS Microbiology Letters*.

[2] Brijwani K, Rigdon A, Vadlani PV (2010). Fungal laccases: Production, function, and applications in food processing. *Enzyme Research*.

[3] Baldrian P (2006). Fungal laccases-occurrence and properties. *FEMS Microbiology Reviews*.

[4] Dwivedi UN, Singh P, Pandey VP, Kumar A (2011). Structure-function relationship among bacterial, fungal and plant laccases. *Journal of Molecular Catalysis B: Enzymatic*.

[5] Mayer AM, Staples RC (2002). Laccase: new functions for an old enzyme. *Phytochemistry*.

[6] Rouhana-Toubi A, Wasser SP, Agbarya A, Fares F (2013). Inhibitory effect of ethyl acetate extract of the shaggy inc cap medicinal mushroom, *Coprinus comatus* (Higher Basidiomycetes) fruit bodies on cell growth of human ovarian cancer. *International Journal of Medicinal Mushrooms*.

[7] Jiang XG, Lian MX, Han Y, Lv SM (2013). Antitumor and immunomodulatory activity of a polysaccharide from fungus *Coprinus comatus* (Mull.:Fr.) Gray. *International Journal of Biological Macromolecules*.

[8] Wang G, Wang J, Fu Y (2013). Systemic treatment with vanadium absorbed by *Coprinus comatus* promotes femoral fracture healing in streptozotocin-diabetic rats. *Biological Trace Element Research*.

[9] Liu X, Zheng X, Zhang J-K (2012). Production of a fibrinolytic enzyme from coprinus comatus YY-20. *Applied Mechanics and Materials*.

[10] Bao S, Teng Z, Ding S (2013). Heterologous expression and characterization of a novel laccase isoenzyme with dyes decolorization potential from *Coprinus comatus*. *Molecular Biology Reports*.

[11] Sun J, Chen QJ, Zhu MJ, Wang HX, Zhang GQ (2014). An extracellular laccase with antiproliferative activity from the sanghuang mushroom *Inonotus baumii*. *Journal of Molecular Catalysis B: Enzymatic*.

[12] Shin K-S, Lee Y-J (2000). Purification and characterization of a new member of the laccase family from the white-rot basidiomycete *Coriolus hirsutus*. *Archives of Biochemistry and Biophysics*.

[13] Gu C, Zheng F, Long L, Wang J, Ding S (2014). Engineering the expression and characterization of two novel laccase isoenzymes from *Coprinus comatus* in *Pichia pastoris* by fusing an additional ten amino acids tag at N-terminus. *PLoS ONE*.

[14] Kellner H, Luis P, Schlitt B, Buscot F (2009). Temporal changes in diversity and expression patterns of fungal laccase genes within the organic horizon of a brown forest soil. *Soil Biology and Biochemistry*.

[15] Sun J, Chen Q-J, Cao Q-Q (2012). A laccase with antiproliferative and HIV-I reverse transcriptase inhibitory activities from the mycorrhizal fungus *Agaricus placomyces*. *Journal of Biomedicine and Biotechnology*.

[16] Laemmli UK, Favre M (1973). Gel electrophoresis of proteins. *Journal of Molecular Biology*.

[17] Hu DD, Zhang RY, Zhang GQ, Wang HX, Ng TB (2011). A laccase with antiproliferative activity against tumor cells from an edible mushroom, white common Agrocybe cylindracea. *Phytomedicine*.

[18] Eggert C, Temp U, Eriksson K-EL (1996). The ligninolytic system of the white rot fungus *Pycnoporus cinnabarinus*: purification and characterization of the laccase. *Applied and Environmental Microbiology*.

[19] Bourbonnais R, Paice MG, Reid ID, Lanthier P, Yaguchi M (1995). Lignin oxidation by laccase isozymes from *Trametes versicolor*and role of the mediator 2,2
′-azinobis(3-ethylbenzthiazoline-6-sulfonate) in kraft lignin depolymerization. *Applied and Environmental Microbiology*.

[20] Coll PM, Fernandez-Abalos JM, Villanueva JR, Santamaria R, Perez P (1993). Purification and characterization of a phenoloxidase (laccase) from the lignin-degrading basidiomycete PM1 (CECT 2971). *Applied and Environmental Microbiology*.

[21] Kojima Y, Tsukuda Y, Kawai Y (1990). Cloning, sequence analysis, and expression of ligninolytic phenoloxidase genes of the white-rot basidiomycete *Coriolus hirsutus*. *The Journal of Biological Chemistry*.

[22] Fukushima Y, Kirk TK (1995). Laccase component of the *Ceriporiopsis subvermispora* lignin-degrading system. *Applied and Environmental Microbiology*.

[23] Giardina P, Aurilia V, Cannio R (1996). The gene, protein and glycan structures of laccase from *Pleurotus ostreatus*. *European Journal of Biochemistry*.

[24] Ben Younes S, Sayadi S (2011). Purification and characterization of a novel trimeric and thermotolerant laccase produced from the ascomycete *Scytalidium thermophilum* strain. *Journal of Molecular Catalysis B: Enzymatic*.

[25] Ng TB (2004). Peptides and proteins from fungi. *Peptides*.

[26] Fang EF, Pan WL, Wong JH, Chan YS, Ye XJ, Ng TB (2011). A new *Phaseolus vulgaris* lectin induces selective toxicity on human liver carcinoma Hep G2 cells. *Archives of Toxicology*.

[27] Zhang G-Q, Wang Y-F, Zhang X-Q, Ng TB, Wang H-X (2010). Purification and characterization of a novel laccase from the edible mushroom *Clitocybe maxima*. *Process Biochemistry*.

[28] Zhu MJ, Du F, Zhang GQ, Wang HX, Ng TB (2013). Purification a laccase exhibiting dye decolorizing ability from an edible mushroom *Russula virescens*. *International Biodeterioration and Biodegradation*.

[29] Wang HX, Ng TB (2006). Purification of a laccase from fruiting bodies of the mushroom *Pleurotus eryngii*. *Applied Microbiology and Biotechnology*.

[30] Zhang G-Q, Chen Q-J, Wang H-X, Ng TB (2013). A laccase with inhibitory activity against HIV-1 reverse transcriptase from the mycorrhizal fungus *Lepiota ventriosospora*. *Journal of Molecular Catalysis B: Enzymatic*.

[31] Wang HX, Ng TB (2004). A new laccase from dried fruiting bodies of the monkey head mushroom *Hericium erinaceum*. *Biochemical and Biophysical Research Communications*.

[32] Tian G-T, Zhang G-Q, Wang H-X, Ng TB (2012). Purification and characterization of a novel laccase from the mushroom pleurotus nebrodensis. *Acta Biochimica Polonica*.

[33] Li M, Zhang G, Wang H, Ng T (2010). Purification and characterization of a laccase from the edible wild mushroom *Tricholoma mongolicum*. *Journal of Microbiology and Biotechnology*.

[34] Min K-L, Kim Y-H, Kim YW, Jung HS, Hah YC (2001). Characterization of a novel laccase produced by the wood-rotting fungus *Phellinus ribis*. *Archives of Biochemistry and Biophysics*.

[35] Edens WA, Goins TQ, Dooley D, Henson JM (1999). Purification and characterization of a secreted laccase of *Gaeumannomyces graminis* var. *tritici*. *Applied and Environmental Microbiology*.

[36] Zhang G-Q, Tian T, Liu Y-P, Wang H-X, Chen Q-J (2011). A laccase with anti-proliferative activity against tumor cells from a white root fungus *Abortiporus biennis*. *Process Biochemistry*.

[37] Guo LQ, Lin SX, Zheng XB, Huang ZR, Lin JF (2011). Production, purification and characterization of a thermostable laccase from a tropical white-rot fungus. *World Journal of Microbiology and Biotechnology*.

